# Rare adverse effects of Electro Convulsive Therapy

**DOI:** 10.1192/j.eurpsy.2023.2161

**Published:** 2023-07-19

**Authors:** C. Licht

**Affiliations:** Psychiatry and Psychotherapy, Paracelsus Medical University, Nuremberg, Germany

## Abstract

**Introduction:**

ECT’s most common side effects are headache, memory impairment, and cardiovascular changes [1]. We report the unusual case of unilateral eyelid swelling and cheek flushing as a side effect of ECT in a depressed but otherwise healthy patient.

**Objectives:**

The patient is a 53-year-old woman with a recurrent depressive disorder for 15 years with a current major depressive episode without psychotic symptoms. With therapy resistance to mirtazapine (60 mg/d) and lithium (675 mg/d; 0.75 mmol/l), an ECT series with a total of seven sessions were performed. Treatment was performed with right unilateral electrode placement according to d’Elia (RUL).

**Methods:**

After the first session (Thymatron IV; energy: 20%; pulse width: 0.5; EEG: 45 s), marked hemifacial erythema and supraorbital lid swelling on the right side were evident. After each of the total seven sessions with adequate seizures in the EEG between 45 to 68 s, hemifacial erythema and supraorbital eyelid swelling were evident on the right side.

**Results:**

Both supraorbital eyelid edema occurred immediately after seizure onset, and hemifacial erythema resolved spontaneously and entirely within 10 minutes after the termination of the respective seizure.

**Conclusions:**

The right side of the face appeared normal before ECT and had no injuries or abnormalities. Trauma, allergic reactions to the anesthetic, or complications of manual ventilation were excluded. In the literature, only one case report with supraorbital eyelid edema [2] and one case report with hemifacial redness of the face [3] after ECT have been described so far. We evaluate the eyelid edema and erythema that occurred in our case as isolated benign complications, most likely due to autonomic activation of facial nerves due to electrical stimulation in RUL.

**Disclosure of Interest:**

None Declared

